# Factors associated with the perception of university professors about academic dishonesty in dental students from two peruvian universities: analysis under multivariable regression model

**DOI:** 10.1186/s12909-023-04281-6

**Published:** 2023-05-02

**Authors:** Marysela Ladera-Castañeda, Flavia León-Málaga, Mary Espinoza-Olórtegui, Miriam Nicho-Valladares, Luis Cervantes-Ganoza, Arturo Verástegui-Sandoval, Fredy Solís-Dante, Miriam Castro-Rojas, César Félix Cayo-Rojas

**Affiliations:** 1 Faculty of Dentistry and Postgraduate School, Research Group “Salud Pública – Salud Integral”, Universidad Nacional Federico Villarreal, Lima, 15001 Peru; 2 School of Stomatology, Universidad Privada San Juan Bautista, Lima, 15066 Peru; 3 Faculty of Stomatology, Universidad Inca Garcilaso de la Vega, Lima, 15084 Peru; 4 Faculdade Do Centro Oeste Paulista, Bauru, Brazil

**Keywords:** Perception, Motivations, Academic dishonesty, Associated factors, Dental students, Professors, Peru

## Abstract

**Background:**

Academic dishonesty is an intentional behavior that transgresses ethics in the teaching-learning process. The present study aimed to evaluate the factors associated with the perception of university professors about academic dishonesty in dental students from two universities in the Peruvian capital.

**Methods:**

This cross-sectional, analytical study evaluated 181 professors from two Peruvian universities between March and July 2022. A validated 28-item questionnaire was used to measure the perceived academic dishonesty of their students. A *logit* model was used to evaluate the influence of the variables gender, marital status, place of origin, academic degree, specialization, academic area, years of teaching experience, scientific publications, ethical training and university of origin, considering a significance level of p < 0.05.

**Results:**

According to the median, professors perceived that their students sometimes had attitudes and motivations to commit academic dishonesty. The professors whose origin was the capital city were twice as likely to perceive dishonest attitudes in dental students as those whose origin was a province (OR = 2.04; 95% CI: 1.06–3.93). University professors in pre-clinical courses were 0.37 times less likely to perceive dishonest attitudes than those teaching in the dental clinic (OR = 0.37; CI: 0.15–0.91). University professors in basic science courses and professors in preclinical courses were 0.43 times (OR = 0.43; CI: 0.19–0.96) and 0.39 times (OR = 0.39; CI: 0.15–0.98) less likely to perceive dishonest motivations in their students compared to university professors in the dental clinic. Gender, marital status, academic degree, specialty, years of teaching experience, scientific publications and ethical training were not found to be influential factors (p > 0.05).

**Conclusion:**

Although all university professors surveyed perceived dishonest attitudes and motivations in their students, university professors from the capital city perceived such attitudes more. In addition, being a preclinical university professor was a hindered factor for perceiving such dishonest attitudes and motivations. It is advisable to implement and constantly disseminate regulations that empower academic integrity as well as to manage a system for reporting misconduct and to make students aware of the impact of dishonesty in their professional training.

## Background

Academic dishonesty encompasses any attitude or behavior that violates the standards set by an institution or scientific community to perform an academic activity [1]. Some examples of student dishonesty could be cheating in exams, pretexts for not taking evaluations, obtaining credit for work in which he/she did not participate and plagiarism in activities, among others [1, 2]. Among the factors that motivate to commit dishonest acts are the overload of academic work, the need to pass or have good grades, the lack of interest, the ease and convenience of access to educational material via the Internet, laziness and poor time management to study and perform academic work, as well as ignorance of the basic rules for developing an academic work [3, 4].

Academic dishonesty is one of the most frequent problems in educational institutions, being persistent over the years and often daily in the activities of students, especially when they have to fulfill some academic assignment [3, 5, 6]. The consequence of this behavior is serious since it not only affects the person who commits such acts but also has a negative impact on educational processes [7, 8].

The evolution of the knowledge society and technology help to locate, research and analyze information quickly and in real time, making it more viable for the student to accomplish different academic tasks [4]. However, just as they provide scientific literacy to the student, they also favor academic fraud by allowing practices such as plagiarism and/or falsification, which generates a complex scenario for the authorities who often cannot identify and control this type of behavior [4, 9–12].

Some studies have reported high frequencies of dishonest attitudes in university students in countries such as Mexico with 80% [3], Canada with values between 50 and 90% [13], and the United States with more than 80% [6]. These offenses to academic integrity produce negative effects on students, graduates and professors by promoting an environment of unfair competition and bad reputation [14, 15].

In general, university professors are aware of the possible dishonest attitudes of some of their students. However, in many cases such attitudes are ignored or not shared with academic managers and/or corresponding authorities, choosing to deal with this issue personally and thus hindering the planning of actions to eradicate the problem in their institutions [2, 16]. On the other hand, the recent Covid-19 pandemic has generated great challenges since most universities have had to adapt methods and strategies to maintain educational quality by creating learning environments conducive to students with academic integrity [17, 18].

The importance of studying the perception of university professors on the attitudes and motivations of dental students for committing academic dishonesty lies in the negative impact this has on the personal and educational level, as it is considered the beginning of corruption and moral dissociation [3, 5]. Likewise, the topic is of special interest in health sciences because students are required to have high levels of ethical principles and values in order to perform optimally when they have the responsibility of caring for a patient [19, 20].

In view of the above, the present study aimed to evaluate the factors associated with the perception of university professors about academic dishonesty in dental students from two universities in the Peruvian capital. The null hypothesis was that there are no factors associated with university professors’ perception of academic dishonesty in dental students.

## Methods

### Study design

This cross-sectional, analytical and questionnaire-based study was written according to the STrengthening the Reporting of Observational studies in Epidemiology (STROBE) guidelines [21]. In addition, it was conducted in professors of the Faculty of Dentistry of the Universidad Nacional Federico Villarreal (UNFV) (located in the Peruvian capital) and the School of Stomatology of the Universidad Privada San Juan Bautista (UPSJB) (based in the capital and with a branch in Ica, a Peruvian province) during March to July 2022.

### Population and selection of participants

The total sample considered was n = 181 (81 professors UNFV and 100 UPSJB professors) since the minimum sample size required was 170 professors according to the statistical program Epidat 4.2 with a formula to estimate a proportion with a total population of N = 201 professors (85 UNFV professors and 116 UPSJB professors) taking into consideration a significance α = 0.05, a precision error of 5%, and an expected proportion p = 0.5 (to obtain the largest possible sample size). The sampling method was stratified randomization considering the following eligibility criteria:

#### Inclusion criteria

- Professors who gave informed consent.
- Professors with academic teaching load at the UNFV School of Dentistry in 2022.
- Professors with academic teaching load of the UPSJB School of Stomatology in 2022.

#### Exclusion criteria

- Professors who did not complete the questionnaire.

### Variables

The dependent variable in the present study was the perception of university professors about academic dishonesty in dental students. The independent variables were gender, marital status, place of origin, academic degree, specialization, academic area, years of teaching experience, scientific publications, ethical training (only if they received at least one ethics course, not at diploma or specialization level) and university of origin [2, 3, 12, 15, 17].

### Validation and application of the instrument

A questionnaire validated by López et al. (2) was used, with 28 items distributed in dimensions D1: Perception about attitude (Items A1 - A14) and D2: Perception about motivations (Items M1 - M14). The answers to each question were on a Likert scale (1: never, 2: rarely, 3: sometimes, 4: often, and 5: always). In addition, questions about sociodemographic factors, training and scientific publications were included. Three experts in stomatological public health and research validated the cross-culturality of the questionnaire, considering pertinence, relevance, clarity, objectivity and timeliness, obtaining an acceptable Aiken V (0.88; 95%CI: 0.84–0.91) (coefficient that allows us to quantify the content validity of the items with respect to a domain, based on the scores of N experiential judges).

Cronbach’s alpha was used to evaluate the reliability of the instrument, obtaining significantly acceptable values for the perception of attitudes with 0.93 (95% CI: 0.91–0.94) and motivations with 0.88 (95% CI: 0.85–0.90). Additionally, 30 professors (15 randomly selected from each institution) were evaluated to check the concordance of the scores obtained at two different times within 10 days and altering the order of the questions to avoid memory bias [22, 23]. The intraclass correlation coefficient (ICC) was very good for perception about attitudes (ICC = 0.98; 95% CI 0.97–0.99) and perception about motivations (ICC = 0.97; 95% CI: 0.94–0.98). The cut-off point for both instruments was determined as the mean of a full score between rarely (28 points) and sometimes (42 points), resulting in 35 points. Furthermore, the accuracy of this cut-off point was validated using Livingston’s K^2^ coefficient, resulting in 0.938 for dishonest attitudes and 0.893 for dishonest motivations, these values being acceptable.

### Procedure

The questionnaire was developed using the Google Forms® virtual platform and was distributed asynchronously to each professor via institutional e-mail or via WhatsApp®. For this purpose, the formal directory of professors was requested from the academic departments of the Faculty of Dentistry of the UNFV and the School of Stomatology of the UPSJB. The invitation to participate was made by the principal investigator (M.L.C) providing her full name, university and contact details such as institutional email and telephone. In some cases, it was necessary to resend the invitation once a week up to a maximum of four times. Upon entering the shared link, professors were automatically directed to the informed consent form and after accepting it, they could enter the questionnaire with the option to answer it only once between March and July 2022. All the researchers had access to the information and the data was stored in a portable digital device with a password to maintain confidentiality. In addition, at the conclusion of the study all the information was destroyed for security reasons. However, the results were previously sent via email to those participants who requested them from the principal investigator. In addition, no incentives were offered for participation in this study.

### Statistical analysis

The Stata v17.0 program (Stata Corp, College Station, TX, USA) was used for data analysis. For descriptive statistics, the relative and absolute frequencies were calculated, as well as the mean and median. For the inferential analysis, the Mann Whitney U test and the Kruskal Wallis H test were used to determine significant differences between variable categories. The Bonferroni post hoc was applied for the variable academic degree and academic area when the Kruskal Wallis test indicated significant differences. To evaluate the influential factors, logistic regression analysis (logit model) was used with the Stepwise method to fit the model. A significance level of 5% (p < 0.05) was considered in all tests.

### Bioethical considerations

By means of approval letter No. PCI6-02-2022, the Ethics Committee of the Faculty of Dentistry of the UNFV authorized the execution of the present study. Likewise, the bioethical principles of non-maleficence, freedom, confidentiality and respect for research on human beings set forth in the Declaration of Helsinki were respected [24]. All participants understood and voluntarily gave informed consent.

## Results

The response rate of UNFV and UPSJB professors was 95.29% and 86.21%, respectively. The average age of the university professors surveyed was 51.06 ± 12.92 years, with 55.8% being male, 57.5% were married or cohabiting, 64.1% were from the Peruvian capital, 67.4% had a Master’s degree, 74.6% had a specialty, 51. 9% taught in the dental clinic, 76.8% had more than 10 years of teaching experience, 60.2% had published at least one scientific article, 55.8% had not received any type of training in ethics and 55.2% worked mainly in a private university. [Table 1]

**Table 1:** Descriptive characteristics of university professors

Variable	Category	Frequency	Percentage
**Gender**	Female	80	44.2
	Male	101	55.8
**Marital status**	Unmarried	77	42.5
	Married or cohabiting	104	57.5
**Origin**	Capital	116	64.1
	Province	65	35.9
**Academic degree**	Bachelor	21	11.6
	Master	122	67.4
	Doctor	38	21.0
**Specialization**	No	46	25.4
	Yes	135	74.6
**Academic area**	Basic sciences	55	30.4
	Preclinical courses	32	17.7
	Dental clinic	94	51.9
**Experience**	< 10 years	42	23.2
	≥ 10 years	139	76.8
**Publications**	No	72	39.8
	Yes	109	60.2
**Ethical training**	No	101	55.8
	Yes	80	44.2
**University**	Public	81	44.8
	Private	100	55.2
**Age**	**Mean**	**Median**	**SD**
	51.06	51.00	12.92

*SD: Standard Deviation*

In general, taking as a reference the median of the responses according to the values assigned on the Likert scale, the surveyed professors perceived that most dental students have sometimes copied from a classmate during the test, used notes during the exam, or shared exam questions with classmates who has not yet taken the exam. The professors also perceived that sometimes students have plagiarized in their activities/assignments and took credit for team assignment in which they did not participate. They also perceived that students sometimes share their work/assignments with students who have not yet taken the course. In addition, the majority of professors perceived that students have never bought/sold exams or had someone impersonate them to take an exam. Similarly, they never perceived students to have unauthorized access to email accounts or systems for dishonest acts, and rarely perceived students to modify medical records/medical notes or research results. [Table 2].

When comparing professors’ perception of students’ dishonest attitudes, it was observed that those from the capital city perceived more dishonest attitudes compared to those from the province regarding items A3 (The student sells/purchases exams), A10 (The student gets credit for a team assignment in which he/she did not participate), A12 (The student invents/alters medical records or medical notes), A13 (The student falsifies his/her participation in clinical activities) and A14 (The student invents results in research papers) (p = 0. 024, p = 0.002, p = 0.025, p = 0.021 and p = 0.001; respectively). Likewise, it was observed that the Doctors perceived more than the bachelors with respect to items A1 (The student copies from a classmate during the exam) (p = 0.008), and more than the masters and bachelors with respect to item A2 (The student shares exam questions with classmates who have not taken the exam) (p = 0.014 and p = 0. 039; respectively), and also the masters perceived more dishonest attitudes than the bachelors with respect to item A8 (The student submits the same work multiple times without the professor’s permission) (p = 0.011). Non-specialist professors perceived more dishonest attitudes towards specialists in relation to items A1 and A2 (p = 0.004 and p < 0.001; respectively). Similarly, clinical professors perceived more dishonest attitudes than preclinical professors for items A8 (p = 0.043) and A9 (The student shares work/assignments with students who have not yet taken the course) (p = 0.028), and more than basic science professors for items A12 (p = 0.023) and A13 (p = 0.001). The professors who have published at least one scientific article perceive more dishonest attitudes compared to those who have not published, in relation to items A3, A12 and A14 (p = 0.023, p = 0.018 and p = 0.023; respectively). Finally, professors who did not receive any ethics course perceived more dishonest attitudes compared to those who did receive at least one ethics course, in relation to items A1, A2 and A6 (Student plagiarizes in activities/assignments) (p = 0.033, p = 0.024 and p = 0.041; respectively), and also professors who teach mainly in private universities perceived more dishonest attitudes compared to those from public universities in relation to item A9 (p = 0.015) [Table 2]

**Table 2:** Comparison of the perception of university professors about dishonest attitudes of dental students

Questions	Total				Gender	Marital status	Origin	Academic degree	Specialization	Academic area	Experience	Publications	Ethical training	University
	Mean	Median	SD		*p	*p	*p	**p	*p	**p	*p	*p	*p	*p
**A1.** The student copies from a classmate during the exam.	2.86	3.00	0.86		0.914	0.870	0.702	0.011**	0.004*	0.574	0.147	0.275	0.033*	0.345
**A2.** The student shares exam questions with classmates who have not taken the exam.	2.76	3.00	0.97		0.063	0.645	0.379	0.009**	< 0.001*	0.908	0.486	0.129	0.024*	0.993
**A3**. The student sells/purchases exams.	1.77	1.00	0.94		0.262	0.058	0.024*	0.472	0.154	0.679	0.224	0.023*	0.440	0.291
**A4**. An external person impersonates a student during an evaluation.	1.41	1.00	0.71		0.174	0.356	0.103	0.140	0.743	0.570	0.443	0.506	0.099	0.913
**A5.** The student uses notes during the exam.	2.55	3.00	1.05		0.392	0.206	0.238	0.449	0.063	0.285	0.576	0.514	0.197	0.180
**A6**. Student plagiarizes in activities/assignments.	2.76	3.00	0.87		0.681	0.074	0.459	0.085	0.323	0.328	0.674	0.459	0.041*	0.109
**A7.** The student plagiarizes in final papers.	2.60	3.00	0.84		0.481	0.497	0.610	0.099	0.389	0.787	0.826	0.095	0.528	0.481
**A8.** The student submits the same work multiple times without the professor’s permission.	2.14	2.00	0.96		0.273	0.553	0.176	0.011**	0.710	0.037**	0.909	0.120	0.139	0.108
**A9.** The student shares work/assignments with students who have not yet taken the course.	2.55	3.00	1.08		0.877	0.563	0.128	0.600	0.894	0.021**	0.901	0.517	0.779	0.015*
**A10.** The student gets credit for a team assignment in which he/she did not participate.	2.51	3.00	0.94		0.471	0.779	0.002*	0.960	0.257	0.099	0.508	0.228	0.579	0.994
**A11.** The student gains access to email accounts or systems in an unauthorized manner.	1.81	1.00	0.94		0.587	0.375	0.056	0.919	0.400	0.407	0.561	0.192	0.452	0.780
**A12.** The student invents/alters medical records or medical notes.	1.99	2.00	0.89		0.943	0.090	0.025*	0.618	0.234	0.019**	0.714	0.018*	0.066	0.139
**A13** The student falsifies his/her participation in clinical activities.	1.97	2.00	0.87		0.891	0.074	0.021*	0.939	0.575	0.001**	0.719	0.141	0.777	0.948
**A14.** The student invents results in research papers.	1.98	2.00	0.92		0.453	0.831	0.001*	0.248	0.072	0.114	0.132	0.023*	0.450	0.512

SD: Standard Deviation; *Based on Mann Whitney U test and **Based on Kruskal Wallis H test, p < 0.05 (significant differences between variable categories). Bonferroni post hoc was applied for the variable “academic degree” and “academic area” when the Kruskal Wallis test indicated p < 0.05. These post hoc p-values were included only in the interpretation.

In general, taking as a reference the median of the responses according to the values assigned on the Likert scale, the professors perceived that most students have sometimes been motivated to commit dishonest acts due to the volume of academic or clinical activities assigned to them, when the assignments or topics do not reinforce their professional training, or when the assignment is not in line with the learning objective. In addition, according to professors’ perceptions, students are sometimes motivated to commit dishonest acts when they realize that assignments are not checked or when they believe they can get a higher grade or when they fear losing a scholarship and feel that the professors allow impunity for these acts. Other reasons that sometimes motivate them are lack of time management and lack of knowledge about the academic integrity regulations. [Table 3]

When comparing the perception of university professors about the motivation of students to commit dishonest acts, it was observed that the married perceived these motivations more than the unmarried in item M11 (Discordance between learning objectives and expectations of the professor) (p = 0.003). The professors from the capital perceived more dishonest motivations than those from the provinces in the items M2 (Poor time management by students), M3 (Ignorance about the academic integrity chapter of the Academic Regulations), M8 (Difficulty for the professor to identify dishonesty), M9 (Perception of impunity for dishonesty), M10 (Lack of follow-up and supervision of assignments or tasks), M11 (Discordance between learning objectives and expectations of the professor), M12 (Poor professor competence in information technology), and M13 (Acceptance of academic dishonesty by peers) (p = 0. 043, p = 0.002, p = 0.021, p = 0.013, p = 0.011, p = 0.005, p = 0.002 and p = 0.028; respectively). In addition, the professors with a doctorate perceived more dishonest motivations than the masters with respect to M6 (The assignments or topics do not reinforce their professional training) (p = 0.049), and it was also observed that the clinical professors perceived these motivations more compared to the basic science professors in relation to the items M1 (Volume of academic or clinical activities), (p = 0.040), M10 (p = 0.013) and M11 (p = 0.002), and compared with preclinical professors in relation to the items M10 (p = 0.015), M11 (p = 0.006) and M13 (p = 0.006). The professors who had not published at least one scientific article perceived more dishonest motivations compared to those who had published, in relation to items M5 (Obtain higher grades) and M14 (The ease of emerging technologies to provide instantaneous retrieval or information storage) (p = 0.011 and p = 0.016; respectively). Finally, it was observed that the professors who taught classes mostly in private universities perceived more dishonest motivations compared to those from public universities, in relation to M4 (Retain the scholarship or financial aid for their studies.) (p = 0.035) [Table 3]

**Table 3:** Comparison of the perception of university professors about dishonest motivations of dental students

Questions	Total				Gender	Marital status	Origin	Academic degree	Specialization	Academic area	Experience	Publications	Ethical training	University
	Mean	Median	SD		*p	*p	*p	**p	*p	**p	*p	*p	*p	*p
**M1.** Volume of academic or clinical activities.	2.60	3.00	0.98		0.684	0.739	0.068	0.808	0.573	0.039*	0.348	0.192	0.322	0.806
**M2.** Poor time management by students.	3.34	3.00	0.95		0.973	0.854	0.043*	0.696	0.524	0.059	0.444	0.724	0.244	0.979
**M3.** Ignorance about the academic integrity chapter of the Academic Regulations.	3.21	3.00	1.14		0.929	0.855	0.002*	0.200	0.122	0.051	0.979	0.184	0.736	0.663
**M4**. Retain the scholarship or financial aid for their studies.	3.01	3.00	1.05		0.383	0.059	0.433	0.408	0.464	0.998	0.797	0.926	0.255	0.035*
**M5.** Obtain higher grades.	3.44	3.00	1.02		0.340	0.875	0.307	0.441	0.152	0.680	0.134	0.011*	0.976	0.824
**M6.** The assignments or topics do not reinforce their professional training.	2.61	3.00	0.94		0.394	0.307	0.511	0.043**	0.786	0.497	0.270	0.942	0.244	0.991
**M7.** Academic honesty is not assessed.	2.93	3.00	1.23		0.049*	0.491	0.426	0.784	0.425	0.131	0.188	0.707	0.539	0.929
**M8.** Difficulty for the professor to identify dishonesty.	2.99	3.00	1.02		0.284	0.057	0.021*	0.873	0.855	0.339	0.822	0.313	0.615	0.517
**M9.** Perception of impunity for dishonesty.	2.91	3.00	1.06		0.983	0.638	0.013*	0.171	0.343	0.212	0.061	0.238	0.260	0.096
**M10.** Lack of follow-up and supervision of assignments or tasks.	2.80	3.00	1.08		0.855	0.054	0.011*	0.332	0.481	0.002**	0.253	0.610	0.485	0.807
**M11.** Discordance between learning objectives and expectations of the professor.	2.68	3.00	1.06		0.809	0.003*	0.005*	0.606	0.699	< 0.001	0.212	0.550	0.894	0.563
**M12.** Poor professor competence in information technology.	2.50	3.00	0.99		0.528	0.472	0.002*	0.296	0.084	0.054	0.174	0.294	0.575	0.076
**M13.** Acceptance of academic dishonesty by peers.	2.59	3.00	1.02		0.482	0.056	0.028*	0.388	0.820	0.009**	0.229	0.537	0.104	0.569
**M14.** The ease of emerging technologies to provide instantaneous retrieval or information storage.	3.39	3.00	1.09		0.815	0.940	0.533	0.774	0.140	0.469	0.642	0.016*	0.745	0.820

SD: Standard Deviation; *Based on Mann Whitney U test and **Based on Kruskal Wallis H test, p < 0.05 (significant differences between variable categories). Bonferroni post hoc was applied for the variable “academic degree” and “academic area” when the Kruskal Wallis test indicated p < 0.05. These post hoc p-values were included only in the interpretation.

According to the adjusted multivariate logistic regression model (logit model) under the stepwise method considering as dependent variables the perception of dishonest attitude and motivations (taking as cut-off point for both variables No [0]: ≤35 points and Si [1]: >35 points), it was observed that professors whose origin was the capital city were twice as likely to perceive dishonest attitudes in students compared to those whose origin was a province (OR = 2.04; CI: 1.06–3.93). It was also observed that professors from pre-clinical courses were 0.37 times less likely to perceive dishonest attitudes than those who taught in the dental clinic (OR = 0.37; CI: 0.15–0.91). Finally, basic science professors and preclinical course professors were 0.43 times (OR = 0.43; CI: 0.19–0.96) and 0.39 times (OR = 0.39; CI: 0.15–0.98) less likely to perceive dishonest motivations in dental students than dental clinic professors. [Table 4]

**Table 4:** Multivariate analysis of influential factors on the perception of university professors about dishonest attitudes and motivations of dental students

Variable	Categories	Perception of dishonest attitudes													Perception of dishonest motivations										
		f	f	Crude model					Adjusted model					f		f	Crude model					Adjusted model			
				OR	95% CI		*p		OR	95%CI		**p					OR	95%CI		p		OR	95%CI		**p
		**Yes**	**No**		**LL**	**UL**				**LL**	**UL**			**Yes**		**No**		**LL**	**UL**				**LL**	**UL**	
**Gender**	Female	32	48	0.78	0.39	1.57	0.489							65		15	1.05	0.46	2.41	0.911					
	Male	41	60	*Ref.*										76		25	*Ref.*								
**Marital status**	Unmarried	33	44	1.35	0.67	2.73	0.396							64		13	1.91	0.79	4.63	0.151					
	Married or cohabiting	40	64	*Ref.*										77		27	*Ref.*								
**Origin**	Capital	54	62	2.19	1.06	4.54	0.034*		2.04	1.06	3.93	0.033**		94		22	1.29	0.57	2.90	0.539					
	Province	19	46	*Ref.*					*Ref.*					47		18	*Ref.*								
**Academic degree**	Bachelor	5	16	1.38	0.32	5.93	0.666							14		7	0.51	0.11	2.45	0.403					
	Master	54	68	2.18	0.90	5.31	0.086							95		27	0.74	0.24	2.24	0.592					
	Doctor	14	24	*Ref.*										32		6	*Ref.*								
**Specialization**	No	14	32	0.57	0.25	1.33	0.194							32		14	0.69	0.28	1.71	0.429					
	Si	59	76	*Ref.*										109		26	*Ref.*								
**Academic area**	Basic sciences	25	30	1.67	0.73	3.78	0.222							39		16	0.43	0.17	1.12	0.085		0.43	0.19	0.96	0.040**
	Preclinical courses	7	25	0.41	0.15	1.14	0.087		0.37	0.15	0.91	0.030**		22		10	0.39	0.14	1.11	0.078		0.39	0.15	0.98	0.046**
	Dental clinic	41	53	*Ref.*					*Ref.*					80		14	*Ref.*					*Ref.*			
**Experience**	< 10 years	17	25	1.31	0.56	3.11	0.534							31		11	0.67	0.24	1.88	0.452					
	≥ 10 years	56	83	*Ref.*										110		29	*Ref.*								
**Publications**	No	24	48	0.65	0.31	1.37	0.261							56		16	1.89	0.79	4.49	0.152					
	Yes	49	60	*Ref.*										85		24	*Ref.*								
**Ethical training**	No	37	64	0.78	0.38	1.59	0.492							76		25	0.74	0.31	1.79	0.507					
	Yes	36	44	*Ref.*										65		15	*Ref.*								
**University**	Public	33	48	1.17	0.57	2.41	0.674							66		15	1.28	0.53	3.06	0.579					
	Private	40	60	*Ref.*										75		25	*Ref.*								
**Age**				1.01	0.98	1.05	0.498										1.00	0.96	1.04	0.894					

f: Absolute frequency; *p < 0.05, significant association according to the crude logistic regression model; **p < 0.05, significant association according to the Stepwise adjusted model.

## Discussion

Academic Integrity has become the most important practice in teaching, since it implies developing values, ethical and moral culture in future generations and thus avoiding corruption and legal problems in society due to dishonest attitudes [25]. It is therefore important to promote academic integrity, especially in the health area, since the professionals who form part of it will put these values into practice when they have the responsibility of caring for the lives of their patients [2, 26]. The present study aimed to evaluate the factors associated with the perception of university professors about academic dishonesty in dental students from two universities in the Peruvian capital.

The median on the perception of dishonest attitudes and motivations indicated that professors have perceived that students sometimes commit acts of academic dishonesty. These results are consistent with Awosoga et al. who reported that post-secondary professors have at some point in their lives perceived some form of academic dishonesty in their students [15]. Likewise, these results are similar to those reported by López et al. who found that the most frequent dishonest acts were the student obtaining credit for work in which he/she did not participate and plagiarism in activities and assignments. They also reported that the main motivators were obtaining higher grades and the facilities offered by new technologies [2]. The results obtained are also similar to those reported by DiPaulo, who found that one of the most frequent dishonest acts perceived by university students was plagiarism of written work [6]. The latter was considered a crime by 68.5% of the students in a study conducted by Castro et al. [4].

It was also found that university professors from the capital city were significantly twice as likely to perceive dishonest attitudes in dental students as professors from the provinces. This is probably due to the fact that professors from the provinces have more confidence in the students, thinking that Peruvian students from the provinces have less access to technological devices, digital tools, virtual platforms and the Internet [27, 28]. This reasoning from a professor’s point of view could hinder the development of dishonest attitudes since it has been reported that greater access to the Internet could favor the student’s temptation to violate academic integrity [17, 29].

In the present study it was observed that professors of preclinical courses were significantly 63% less likely to perceive dishonest attitudes than those professors who taught in the dental clinic. It was also observed that professors of basic sciences and professors of preclinical courses were significantly 57% and 61%, respectively, less likely to perceive dishonest motivations in dental students than those professors who taught in the dental clinic.

This discrepancy may be due to the fact that professors in clinical areas, given the lack of face-to-face practice due to the pandemic, increased clinical reasoning activities in virtual teaching [30], leading to greater interaction with students. Professors constantly sought to demonstrate the application of acquired knowledge through the development of skills, abilities and attitudes in each competency [31]. This probably allowed them to better identify some dishonest attitudes and motivations when evaluating clinical activities, since in this area it is common that the assignments show a lack of information and difficulties in the analysis and interpretation of data [7]. This argument is reinforced by other studies that reported the use of virtual resources as a possible origin of questionable academic behavior by students. These behaviors may vary according to the subject, the topic and the strategies employed for the development of the practices [32, 33].

The present study aimed to survey professors to determine their perceptions about the motivations for dishonest attitudes since most studies evaluate the perception of students about academic dishonesty [3, 13, 34, 35]. However, students and professors have different perceptions about dishonest acts and the seriousness of such infractions [36]. Therefore, it is essential to highlight the role of professors as the main responsible for the student’s formative process and being in direct contact with them to develop a fundamental role in the promotion of individual responsibility, the transmission of values, personal and professional ethics in each subject, as well as in the reporting and prevention of dishonest behaviors in their students and in their own professional practice [2, 3, 35, 37]. The contribution of this study is relevant because it allows the identification of acts of academic dishonesty in higher education institutions, which have increased in the context of the pandemic and may have a negative impact on society in the medium or long term [2, 5, 38]. It is necessary to train professors and provide them with virtual tools that allow them to identify academic dishonesty in the area of health sciences, since these disciplines require putting into practice moral values and professional ethics that could later have an impact on the quality and safety of patients during their care [2, 19, 39].

Among the limitations of the present study was the inability to survey professors in person, since at the time of the survey Peru was in a new wave of Covid-19 [40, 41] and the majority of classes were virtual. Nor was it possible to make a comparison of the perception and motivations about dishonest attitudes among students and professors. Finally, the cross-sectional design of the present study did not allow us to assess the variation and durability of the perception of university professors about the motivations and attitudes of students to commit academic dishonesty.

Based on the results obtained, it is recommended that longitudinal studies be designed to evaluate the impact of educational interventions on academic integrity in university students. Likewise, it is recommended that educational institutions promote a culture of integrity and honesty in all their subjects, which will help the integral formation of students. It is also suggested that educational institutions carry out collaborative work between students, professors and administrators to prevent acts of academic dishonesty, as well as to establish institutional policies that promote academic integrity and facilitate the inclusion of effective methods of reporting, follow-up and/or sanctioning of dishonest acts [2, 20, 26, 36, 42].

## Conclusion

Although all university professors surveyed perceived dishonest attitudes and motivations in their students, university professors from the capital city perceived such attitudes more. In addition, being a preclinical university professor was a protective factor for perceiving such dishonest attitudes and motivations. These dishonest motivations were little perceived by basic science professors. It is advisable to implement and constantly disseminate regulations that empower academic integrity as well as to manage a system for reporting misconduct and to make students aware of the impact of dishonesty in their professional training.

## Data Availability

All data analyzed during this study are available from the principal author on reasonable request (mladera@unfv.edu.pe).

## Abbreviations

CI: Confidence interval
OR: Odds ratio
SD: Standard Deviation
STROBE: Strengthening the Reporting of OBservational studies in Epidemiology
SPSS: Statistical Package for the Social Sciences
UNFV: Universidad Nacional Federico Villarreal
UPSJB: Universidad Privada San Juan Bautista.

## References

[1] Guerra L (2017). Professionals training and the academic dishonesty. Rev Educ Valores.

[2] López D, Eraña I, Segura N, Piedra I, Díaz J, López M (2020). Faculty perceptions of dishonesty on medical students: prevalence, motivations and implications. Educ Med.

[3] Diez-Martínez E (2015). Deshonestidad académica de alumnos y profesores: Su contribución en la desvinculación moral y corrupción social. Sinéctica.

[4] Castro Y, Yoplac-Lopez B, Carpio-Tello A, Sihuay-Torres K, Cósar-Quiroz J (2018). Perception of academic plagiarism by dentistry students. Educ Med.

[5] Díaz R, Méndez MA, Zapata DP, Gurrola A (2022). Academic dishonesty as perceived by graduate students. Cienc lat.

[6] DiPaulo D (2022). Do preservice teachers cheat in college, too? A quantitative study of academic integrity among preservice teachers. Int J Educ Integr.

[7] Eraña IM, López DM, Díaz R, López MV (2018). This does not happen at our school: perceptions about academic dishonesty with medical students. Educ Méd.

[8] Reskala FJ (2020). Nuevos comportamientos de deshonestidad académica en estudiantes mexicanos: Un estudio exploratorio. Informes Psicol.

[9] Thomas J, Jeffers A (2020). Mobile eye tracking and academic integrity: a proof-of-concept study in the United Arab Emirates. Acc Res.

[10] Novick PA, Lee J, Wei S, Mundorff EC, Santangelo JR, Sonbuchner TM (2022). Maximizing Academic Integrity while minimizing stress in the virtual Classroom. J microbiol biol.

[11] Baran L, Jonason PK (2020). Academic dishonesty among university students: the roles of the psychopathy, motivation, and self-efficacy. PLoS ONE.

[12] Kiekkas P, Michalopoulos E, Stefanopoulos N, Samartzi K, Krania P, Giannikopoulou M, Igoumenidis M (2020). Reasons for academic dishonesty during examinations among nursing students: cross-sectional survey. Nurse Educ Today.

[13] Eaton SE, Crossman K, Edino RI (2019). Academic Integrity in Canada: an annotated bibliography.

[14] Gómez AI, Pinto BJ (2018). La integridad académica: el dilema de la formación médica. Revista Educación y Desarrollo Social.

[15] Awosoga O, Nord CM, Varsanyi S, Barley R, Meadows J (2021). Student and faculty perceptions of, and experiences with, academic dishonesty at a medium-sized canadian university. Int J Educ Integr.

[16] Gallent C. Tello Percepción docente sobre el ciberplagio académico en el marco de la enseñanza universitaria online. Valencia: Congreso In-Red. 2019;1:1716-28. 10.4995/INRED2019.2019.10383

[17] Alessio HM, Messinger JD (2021). Faculty and Student perceptions of Academic Integrity in Technology-Assisted learning and testing. Front Educ.

[18] Academia de Integridad Académica. La integridad académica en tiempos de pandemia. 2020. Available from: https://academicintegrity.org/resources/blog/60-2020/november-2020/222-la-integridad-academica-en-tiempos-de-pandemia

[19] Orellana S, Alucema A, Araya P, Segovia E, Guevara Z, Fernández E (2022). Perception on the behaviors that relate to academic integrity in pharmacy students at a chilean university. Educ Med.

[20] Malik M, Mahroof A, Ashraf M (2021). Online University Students’ perceptions on the awareness of, reasons for, and solutions to Plagiarism in Higher Education: the development of the AS&P model to Combat Plagiarism. Appl Sci.

[21] Von Elm E, Altman DG, Egger M, Pocock SJ, Gøtzsche PC, Vandenbroucke JP (2008). The strengthening the reporting of Observational Studies in Epidemiology [STROBE] statement: guidelines for reporting observational studies. Gac Sanit.

[22] Cayo-Rojas CF, Soto-Castro L, Castro-Mena M, Medrano-Colmenares S, López-Gurreonero C, Córdova-Limaylla N (2022). Level of knowledge about metalloproteinases in dental students close to graduate from three universities in peruvian capital city. Eur J Dent Educ.

[23] Allen-Revoredo C, Ladera-Castañeda MI, Córdova-Limaylla NE, Briceño-Vergel G, Cervantes-Ganoza LA, Cayo-Rojas C (2022). Knowledge, attitudes, and practices on oral health prevention associated with sociodemographic factors of adolescent students from a peruvian-swiss educational institution. J Int Oral Health.

[24] World Medical Association (2013). World Medical Association Declaration of Helsinki: ethical principles for medical research involving human subjects. JAMA.

[25] Cortés-Nájera M (2022). Academic Integrity, a challenge in times of pandemic. Con-cienc bol cient esc preparatoria no 3.

[26] Sousa RN, Conti VK, Salles AA, Mussel ID (2016). Desonestidade acadêmica: reflexos na formação ética dos profissionais de saúde. Rev Bioét.

[27] De la Asunción IN, Ypanaque IL, Callacondo VM (2022). Brecha digital y la problemática del derecho a la educación en zonas rurales durante el estado de emergencia. Derecho.

[28] Cruz JL (2022). ICTs and their impact on rural education: reality, challenges and perspectives to achieve equitable education. Cienc lat.

[29] Espiñeira EM, Muñoz JM, Gerpe EM, Castro MD (2021). Cyber-plagiarism as digital support for the submission of academic writing. Comunicar.

[30] Núñez-Cortés JM, Reussi R, García M, Falasco S (2020). COVID-19 and medical education: a look to the future. Latin American Medical Education Forum (FIAEM). Educ Med.

[31] Torres-Contreras C, Bravo-Gómez M (2021). Professors’ perception about evaluation of clinical nursing practice. Educ Med Super.

[32] Porto AM, Mosteiro MJ, Gerpe EM (2022). Lorenzo Rey Álvaro. University students’ perspectives on Plagiarism during the COVID-19 pandemic. Revista Fuentes.

[33] Gómez MA, García González JI, Benítez FE (2022). Academic dishonesty practices in digital distance education, perceptions of students and teachers while covid-19 pandemic. Universidad Católica de Pereira.

[34] Arévalo-Avecillas D, Padilla-Lozano C, Perez-Villamar J, Matute-Fernández M. (2019). Diferencias en las Actitudes Frente a la Deshonestidad Académica entre Estudiantes de Pregrado de Administración y de Economía en la provincia del Guayas, Ecuador. Form. Univ. 2019;12(5):41–50. 10.4067/S0718-50062019000500041

[35] Maring J, Vail M, Wright KA, Tebbenhoff B, Canova K, Costello E. (2018). Attitudes Toward Academic Dishonesty in Health Profession Students. J Allied Health 2018;47(4):97–103. 30508844

[36] Anggreini YS, Prabandari YS, Prihatiningsih TS. (2017). The Preceptions of students and Teachers about The Level of The Sanctions for academic Integrity Violans: An Explanatory Sequential Design Study in a Nursing Education Program. J. Pendidik. Kedokt. Indones 2017;6(2):84–92. 10.22146/jpki.32248

[37] Abusafia AH, Roslan NS, Mohd Yusoff D, Mat Nor MZ (2018). Snapshot of academic dishonesty among malaysian nursing students: a single university experience. J Taibah Univ Sci.

[38] Vásquez-Rocca L, Cuba C, Vásquez C (2022). Awareness, prevalence, and assessment of dishonest practices in university students of arts and humanities and Health Sciences. In Ed.

[39] Devine CA, Chin ED, Sethares KA, Asselin ME. (2021). Nursing Student Perceptions of Academic and Clinical Integrity. Nurs. Educ. Perspect. 2021;42(4):221–226. 10.1097/01.NEP.0000000000000806 33813537

[40] Center for Systems Science and Engineering (CSSE) at Johns Hopkins University (JHU). COVID-19 Dashboard Peru. 2022. Available from: https://www.arcgis.com/apps/dashboards/bda7594740fd40299423467b48e9ecf6.

[41] Cayo-Rojas C, Córdova-Limaylla N, Ladera-Castañeda M, Briceño-Vergel G, López-Gurreonero C, Castro-Mena M (2022). Psychological distress facing the COVID-19 pandemic in dental interns from the peruvian capital: a cross-sectional study under a multivariable regression model. Front Public Health.

[42] Cayo-Rojas C, Paucar-Rodríguez E, Miranda-Dávila A (2022). The role of university teachears facing academic dishonesty from their students. Rev Cuba Inf Cienc Salud.

